# Maintaining your voice as an underrepresented minority during the peer review process: A dialogue between author and mentor

**DOI:** 10.1007/s40037-022-00707-x

**Published:** 2022-03-18

**Authors:** Monnique Johnson, Abigail Konopasky

**Affiliations:** 1 School of Medicine, Uniformed Services University of the Health Sciences, Bethesda, MD USA; 2 Center for Health Professions Education (CHPE), Uniformed Services University of the Health Sciences, Bethesda, MD USA; 3 The Henry M Jackson Foundation for the Advancement of Military Medicine, Bethesda, MD USA

## Background

On my journey as a novice researcher, I (MJ) wrote and submitted a perspective piece based on my unique experience as a Black Woman in Medicine and the intersectionality of that identity. This writing process for me was vulnerable and emotional. I submitted my piece to multiple academic medical journals throughout the peer review process and ultimately received a revise and resubmit. Throughout, I received comments from reviewers and editors, which in itself was a further experience of vulnerability for me. Below, my mentor and I share our perspectives on this process.

## Author view

My perspective piece started as a personal application statement to a Health Professions Education program. I had no intention of publishing it. It was not until one of my mentors (AK) encouraged me that others *needed *to hear what I had to say and that they could learn from my voice that I gained confidence to consider publication. I began to feel that I, a medical student, had the expertise and voice to teach a community of well-educated scholars. That confidence was powerful. It was a driving force to continue building on the shell of my personal statement, getting it ready for submission. Fleshing out my feelings, reactions and thoughts to some of the negative experiences and trauma I had previously endured throughout medical school was a painful process. In writing these experiences, I had to relive unpleasant moments in great detail in order to explain them in a way others could understand. After months of editing back and forth with AK, I was ready to submit.

### The peer review

After the disappointment from not seeing the word “accepted” faded away, I began to feel triggered as I read the peer reviewers’ suggestions. My confidence in the value of my voice plummeted immediately as I realized that addressing these suggestions would mean going deeper into the details of my medical school experiences, making them all fresh again. I would have to reopen old wounds, relive my trauma, and find a way to yet again validate my experience by grounding it in the work of others to satisfy peer reviewers and get published. The peer reviews, while certainly part of the process and well intentioned, were assumptive of my experience and almost discouraged me from resubmitting because it felt too painful to edit.

Overall, the peer reviews were positive, but the framing suggested that the reviewers were unsatisfied with how I explained my experience. Thus, to help me flesh out my piece, they* told *me what I was experiencing and what *I meant *to say. I was given examples of what the peer reviewers learned from their own experiences and what they thought *I* should have learned. I was offered references to other Black experiences to show me how I should frame *my *experience. The suggestion was that to incorporate these references would make my piece “stronger.” In other words, I had to frame my experience through previous literature to be “acceptable” for academia; I needed more published experiences to co-sign *my own *in order for it to be validated. The suggestions made me feel that I needed to use the experiences of others—other people who studied Black experiences, or other Black researchers, not even Black women in medicine—to make the claim that I experienced what I experienced. This reinforced the idea that *my *story was not good enough, that if it did not happen in the literature then it is not real or not relevant enough to be believed. This was ironic because the piece was about the exact opposite. The messages that I took from these suggestions, although not directly spoken, were 1) I was not being heard—the peer reviewers were placing their assumptions and perspectives on me—and 2) the message that rang the loudest, “*you* are not enough,” “your *story *is not enough,” “your *voice *is not enough.”

All of this, I believe, carried no malicious intent. I think the reviewers were trying to be sensitive and intentional. In fact, I’m sure they thought they were. However, intent without proper execution can have fatal results. Thankfully, due to the strong mentorship I had, that was not the case. I don’t say this to suggest that peer reviewers or editors should censor themselves; I say it because I initially did not have the confidence to put my voice on a published platform—after all, it was just my experience. But I gained confidence to get published through mentorship when AK saw value in what I was saying and encouraged me that what I had to say was important.

## Mentor view

After seeing the phrase, “revise and resubmit,” I was elated that MJ’s (one of my mentees) personal piece about her experiences was being moved to the next stage of publication. To me, a white woman with a solid position in the academic world and experience publishing, the editorial and reviewer suggestions seemed like a relatively minor ask, a victory. Even after hearing her concerns, I attributed Monnique’s reaction to the pain any novice writer feels when her words are hacked apart. Despite work I was doing to dismantle my white racial frame, I initially approached MJ through this frame, dismissing her experience.

As we sat down and reviewed the comments together, however, I began to see the ways her experience was being dismissed and devalued, subordinated to voices from “the literature” that preceded her. I also began to see my *own* voice in those comments, down to particular words or phrases *I* had used in prior reviews. I saw how, even with good intent, I had been complicit in subordinating other authors’ voices. Working through the revision process through *Monnique’s* lens, rather than my own, I was able to help her process the comments. I offered emotional support and practical advice for addressing the reviewers’ concerns, working to keep *her* voice front and center.

## Recommendations

We don’t want other underrepresented minority (URM) authors to experience the vulnerability and loss of confidence MJ did, particularly when submitting pieces about personal experiences. If it were not for the support system that MJ had to encourage her and remind her that her voice needed to be heard, guiding her in a way that was able to do that *and *address the points of the reviewers, her experience would be unavailable for others to learn from. Based on this experience, we offer recommendations for authors, mentors, reviewers and editors to support the voices of URM scholars with intentionality and sensitivity.

*Authors*: Seek a supportive mentor who will help you maintain the integrity of your own voice and navigate reviewer comments. Ask trusted friends to help you emotionally process any racial trauma that might be arising.

*Mentors*: Try to see the comments through your mentee’s lens—what might they be experiencing that you wouldn’t? Help your mentee respond to the suggestions while keeping the integrity of their voice.

*Reviewers*: Do not assume you know what the author is “trying” to say: note only what you see through statements like, “I read this as …” Value the author’s experience and be intentional about what role other voices from the literature would play in the piece and whether they are needed.

*Editors*: Offer reviewers different instructions for non-research pieces, particularly those discussing potential racial trauma. As an editor, consider sponsoring a scholar of color through the publishing process.

